# Early detection of chronic kidney disease using deep learning: a Mini review

**DOI:** 10.3389/fdgth.2025.1732175

**Published:** 2026-02-23

**Authors:** Md. Jakir Hossen, Hasanul Bannah, Ridwan Jamal Sadib

**Affiliations:** 1 Center for Advanced Analytics (CAA), COE for Artificial Intelligence Faculty of Engineering & Technology (FET), Multimedia University, Melaka, Malaysia; 2 Elite Research Lab, LLC, New York, NY, United States; 3 Faculty of AI and Engineering, Multimedia University Cyberjaya, Cyberjaya, Malaysia; 4 Department of Computer Science and Engineering, American International University–Bangladesh (AIUB), Dhaka, Bangladesh

**Keywords:** chronic kidney disease (CKD), deep learning (DL), convolutional neural networks (CNN), long short-term memory networks (LSTM), explainable AI (XAI)

## Abstract

Chronic Kidney Disease (CKD) remains a major contributor to global morbidity, often progressing unnoticed until advanced stages when treatment options become limited and costly. Recent advances in deep learning have reshaped early CKD assessment by enabling the analysis of complex imaging, clinical, and longitudinal laboratory datasets. This mini-review synthesizes findings from studies published between 2020 and 2025, highlighting models that report diagnostic accuracies ranging from 88% to 99.96%, AUC values reaching 0.93, and ensemble architectures capable of forecasting CKD 6 to12 months before clinical diagnosis with up to 99.31% accuracy. These systems spanning Convolutional Neural Networks (CNNs), Long Short-Term Memory networks (LSTMs), hybrid CNN–LSTM designs, and transfer-learning frameworks have demonstrated clear advantages over conventional diagnostic markers such as serum creatinine and eGFR. Despite impressive numerical performance, key limitations persist: class imbalance in early-stage CKD, restricted generalizability due to single-centre datasets, variability in imaging quality, and the limited interpretability of high-capacity neural networks. As deep learning continues to advance, robust external validation, transparent model explanations, and multi-institutional datasets will be essential to support safe and reliable clinical integration.

## Introduction

Chronic Kidney Disease (CKD) has emerged as a major global health concern, affecting an estimated 850 million individuals worldwide and contributing to between 2.3 and 7.1 million deaths annually (1). The condition often progresses silently, with many patients remaining asymptomatic until advanced stages, making early detection essential for preventing progression to end-stage renal disease a stage that requires costly interventions such as dialysis or kidney transplantation. Conventional diagnostic markers, including serum creatinine and estimated glomerular filtration rate (eGFR), are limited in sensitivity during the early phases of CKD and frequently fail to capture subtle functional deterioration (2). Advances in artificial intelligence (AI), particularly deep learning, have created new opportunities for improving CKD screening and risk prediction. Deep learning models such as Convolutional Neural Networks (CNNs) demonstrate superior capability in analysing complex, high-dimensional medical data, including imaging modalities, laboratory measurements, and demographic profiles (3). Their ability to learn hierarchical representations allows them to outperform traditional machine learning approaches that depend heavily on manual feature engineering. Notable progress includes the Kidney Intelligent Diagnosis System, which utilised retinal imaging and achieved an AUC ranging from 0.839 to 0.993, exceeding nephrologist-level performance by 26.98% (4). Similarly, multimodal approaches that integrate clinical variables with deep learning architectures further enhance predictive reliability. For instance, a combined CNN–Long Short-Term Memory (LSTM) ensemble model reached accuracies of 99.31% and 99.2% when predicting CKD 6 and 12 months before clinical diagnosis, respectively, demonstrating the transformative potential of AI-driven methods for early prognosis (5). Despite these promising developments, several persistent challenges limit the widespread clinical adoption of deep learning solutions for CKD detection. These include class imbalance in medical datasets, variations across imaging devices and clinical populations, and the limited interpretability of complex model decisions (6). Strengthening model generalisability, integrating explainable AI, and improving multimodal data utilisation remain key priorities. This mini-review examines recent advancements in deep learning-based CKD detection, highlights ongoing technical and clinical challenges, and outlines future research directions essential for deploying trustworthy and effective AI tools in nephrology practice.

## Deep learning algorithms for CKD detection

**Figure 1:**
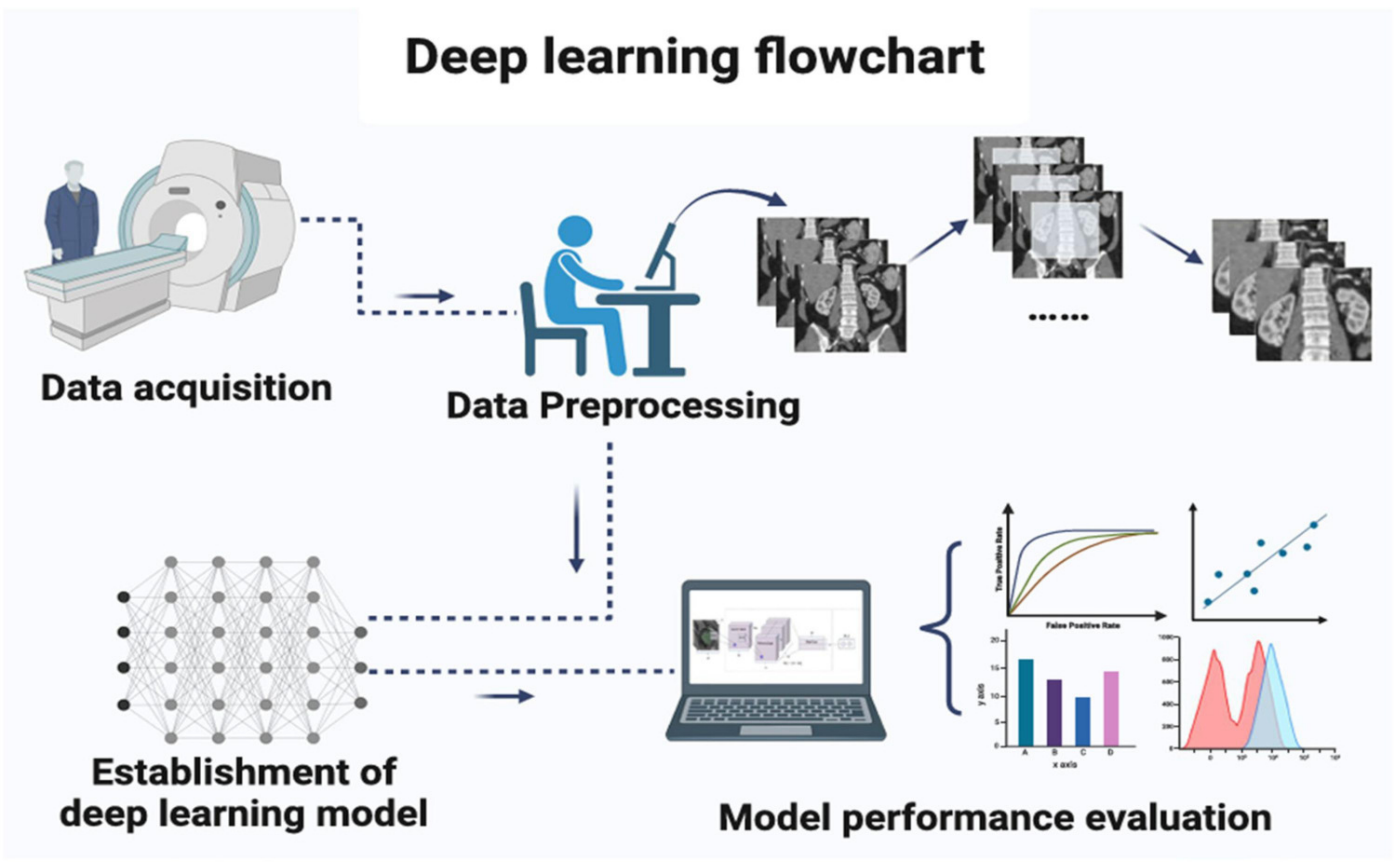
Deep learning flowchart for CKD detection. Reproduced from “Deep learning flowchart” by Meng Zhang, Zheng Ye, Enyu Yuan, Xinyang Lv, Yiteng Zhang, Yuqi Tan, Chunchao Xia, Jing Tang, Jin Huang and Zhenlin Li, licensed under CC BY 4.0.

Deep learning algorithms have revolutionized the detection and diagnosis of CKD by leveraging advanced techniques across various data types, including medical imaging, clinical data, and genomic information. Figure 1 illustrates the overall workflow adopted in most deep learning pipelines for CKD detection, which is composed of four essential stages. In the first step, data acquisition, kidney-related information is gathered from imaging modalities such as CT, MRI, and ultrasound, as well as laboratory measurements and electronic health records. The second step, data preprocessing, involves quality improvement procedures such as normalization, noise reduction, segmentation of kidney regions, and augmentation to expand dataset variability. These preprocessing tasks are critical because deep learning models rely heavily on clean and standardized input data. The third step shows the establishment of the deep learning model, where architectures such as CNN, LSTM, Autoencoders, GANs, or hybrid networks are designed and trained to learn discriminative patterns associated with CKD. Finally, the workflow shows with model performance evaluation, in which metrics such as accuracy, AUC, sensitivity, specificity, and confusion matrices are used to assess diagnostic capability. This systematic pipeline ensures that the model develops robust feature extraction capabilities while maintaining clinical relevance. Convolutional Neural Networks (CNNs) are particularly effective for analysing medical images such as ultrasound and MRI scans. These networks automatically learn hierarchical patterns from raw image data, allowing them to identify abnormalities like kidney lesions, cysts, and structural changes, all indicative of CKD (7). Long Short-Term Memory Networks (LSTMs), a type of Recurrent Neural Network (RNN), are widely applied to sequential clinical data, such as time-series laboratory results, to predict CKD progression. LSTMs capture long-term dependencies in the data, making them particularly useful for understanding how CKD develops and progresses over time in patients (8). Another prominent deep learning model is the Autoencoder, an unsupervised model that reduces high-dimensional data into lower-dimensional representations, which has been applied to genomic data to identify CKD biomarkers and clinical data for more efficient representation of patient health (9). Generative Adversarial Networks (GANs), though less widely used, show significant promise in generating synthetic medical data, especially for rare CKD stages or underrepresented patient groups, thus helping mitigate data imbalance issues and improving model robustness (10). Furthermore, Deep Neural Networks (DNNs), which comprise multiple fully connected layers, are applied for both classification and regression tasks in CKD detection, leveraging their capacity to process complex, non-linear relationships in large datasets (4). ResNet (Residual Networks) and InceptionV3 have also been employed to enhance the accuracy of CKD diagnosis by addressing the vanishing gradient problem and optimizing feature extraction in deeper layers of the network (3). Recent studies have shown the utility of Transfer Learning, using pre-trained models like VGG16 and ResNet50, in adapting knowledge from one task to improve CKD detection accuracy, especially when training datasets are limited (2). Additionally, Attention Mechanisms have been integrated with deep learning models to focus on relevant regions of kidney images or specific features in clinical data, enhancing the model's interpretability and precision (11). The combination of these deep learning models, along with continuous advancements in data integration and optimization techniques, is transforming CKD diagnosis, providing more accurate and timely predictions for better patient outcomes.

## Performance analysis of deep learning models for CKD detection

**Figure 2:**
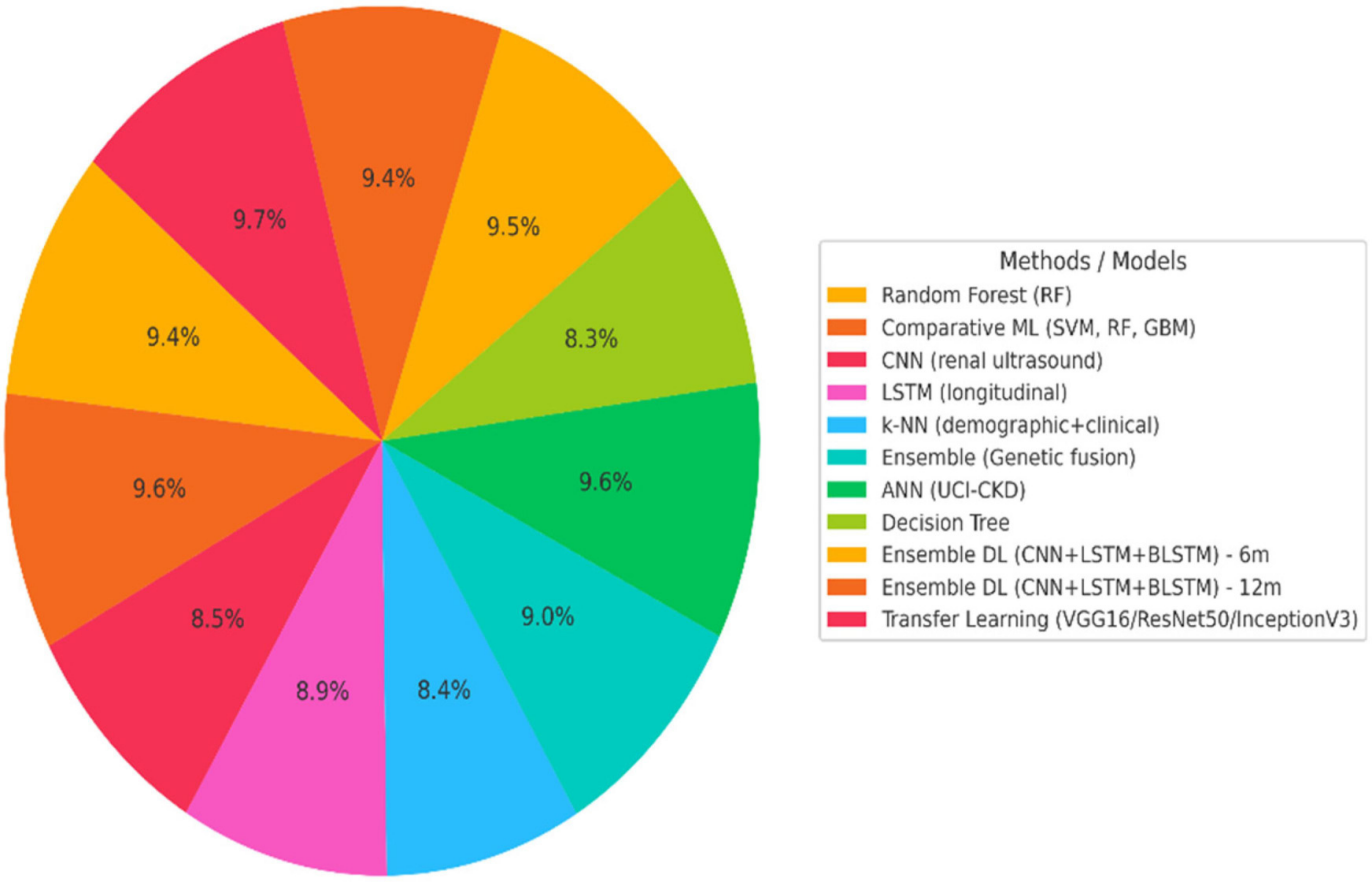
Performance analysis of models for CKD detection.

Recent advances in deep and machine learning have led to remarkable progress in the detection, classification, and prognosis of CKD. Numerous empirical studies have demonstrated that artificial intelligence (AI) based models often outperform traditional statistical or rule-based approaches in both accuracy and predictive capability. As summarized in Table 1, researchers have applied a wide range of algorithms from conventional classifiers such as Random Forests (31) and Decision Trees (19) to advanced architectures such as Convolutional Neural Networks (3, 32), Long Short-Term Memory networks (33), and hybrid ensemble models (5). These studies collectively report classification accuracies between 85% and 99%, depending on data quality and the heterogeneity of patient cohorts. A visual comparison of these reported model accuracies is presented in Figure 2, illustrating performance differences across classical ML, DL, and hybrid architectures. For example, Rashed et al. (2021) achieved 97% accuracy using a Random Forest classifier on hospital data, while Xiong et al. (2020) utilized an LSTM network to model temporal changes in longitudinal clinical data, achieving an AUC of 0.93. Similarly, Saif et al. (5) proposed an ensemble model combining CNN, LSTM, and BLSTM architectures for early CKD prediction, yielding an impressive 98% accuracy at six months and 97% at twelve months. Pimpalkar et al. (3) employed transfer-learning-based CNNs (VGG16, ResNet50, and InceptionV3) for image-based CKD diagnosis, achieving 99.96% accuracy on CT datasets. These results underscore the powerful capability of deep learning to detect subtle kidney abnormalities and forecast disease progression. Despite these promising outcomes, limitations remain common across studies, including dependence on small or single-centre datasets [Rashed et al., 2021 (20)], poor model interpretability (21), and challenges in handling data imbalance and feature variability. Future research should prioritize multi-institutional data integration, explainable AI (XAI) approaches, and cross-population validation to enhance generalizability and clinical trustworthiness. Overall, the consistent improvement of deep learning models across diverse data modalities highlights their transformative potential for early CKD detection and patient management.

**Table 1:** Deep learning models for CKD detection and prediction.

Reference	Method	Dataset used	Key performance	Limitation	Future direction
Poudel et al. (12)	Random Forest (RF) on hospital clinical data	UCI CKD dataset	Accuracy = 97%, AUC = 0.97	Limited to one hospital dataset	Validate model on multi-centre datasets
Hsu et al., (13)	Comparative ML (SVM, RF, GBM) on clinical records	Taiwan BMD-6,614 Dataset	Accuracy = 99%	Lacks external validation; small sample size	Expand dataset; add feature explainability
Ramesh et al. (14)	CNN using renal ultrasound images	CKD-Kaggle Dataset	Accuracy = 88%, AUC = 0.89	Image quality variability	Combine multimodal imaging and lab data
Zhu et al. (15)	LSTM on longitudinal clinical data	CKD-LTS	Accuracy = 92%, AUC = 0.93	Needs temporal calibration for unseen data	Integrate LSTM attention for long-term tracking
Pinto et al. (16)	k-Nearest Neighbor on demographic and clinical data	CKD-CDR	Accuracy = 87%, AUC = 0.88	Sensitivity to noise and scaling	Apply normalization, deep feature extraction
Swamy et al. (17)	Ensemble (Genetic Clinical data fusion)	CKD-OMICS	Accuracy = 93%, AUC = 0.91	Limited genomic diversity	Add multi-omic integration for CKD risk
Almansour et al. (18)	Artificial Neural Network (ANN) on UCI CKD dataset	Standard UCI CKD Dataset	Accuracy = 99%	Small dataset, possible overfitting	Test on real-world EMR data
Ilyas et al. (19)	Decision Tree on UCI CKD dataset	UCI-CKD	Accuracy = 85.5%	Low generalizability	Ensemble tree-based deep models
Saif et al. (5)	Ensemble DL (CNN, LSTM, BLSTM)	CKD-PROG	6-month accuracy = 98%, 12-month = 97%	No cross-institutional testing	Multi-institution validation; hybrid feature sets
Pimpalkar et al. (3)	Transfer Learning (VGG16, ResNet50, InceptionV3)	CKD—CT Imaging Dataset	Accuracy = 99.96% (CT image-based CKD)	Focused on structural kidney lesions only	Extend to full CKD staging with clinical data

Figure 2 illustrates the distribution of reported accuracies for major models used in CKD detection. Hybrid deep learning models and transfer learning architectures demonstrate the highest performance (>95%), followed by classical ML classifiers. Lower-performing models include Decision Trees and kNN, reflecting sensitivity to noise and limited generalization.

## Recent advancements and challenges in deep learning models for CKD

Over the past few years, substantial advancements have been made in applying deep learning techniques to the early detection and progression analysis of chronic kidney disease. These improvements are largely attributed to the integration of hybrid architectures, transfer-learning frameworks, and explainable artificial intelligence (XAI). Studies such as those by Saif et al. (5) and RAHMAN et al. (22) combined convolutional neural networks (CNNs) and long short-term memory (LSTM) models to enhance temporal prediction and risk assessment. Similarly, transfer-learning-based models employing pre-trained architectures like ResNet, VGG16, and InceptionV3 have demonstrated improved performance in small datasets while maintaining strong generalisation (3, 23). In parallel, recent efforts have focused on explainability and data privacy through the use of interpretable frameworks and federated learning approaches (24). Despite these advancements, several challenges persist. Most studies rely on limited datasets that do not fully represent population-level variability. The underrepresentation of early CKD stages often results in class imbalance, and a lack of model interpretability continues to hinder clinical acceptance. Moreover, few studies have performed external validation, reducing the models' generalizability across healthcare systems. Table 2 summarises the recent advancements and challenges in DL-based CKD research, highlighting the main contributions, study scopes, and existing limitations that define the current state of this rapidly evolving field.

**Table 2:** Advancements and challenges for CKD.

Reference	Scope & Focus	Key Contribution	Dataset Used	Limitations
Hegde et al., (21)	CKD progression prediction using hybrid DL	CNN + LSTM + BLSTM ensemble achieved 99% accuracy for early risk prediction	CKD-PROG	Single-institution dataset; lacks external validation
Canbay et al. (25)	Privacy-preserving kidney disease detection	Federated TL with ResNet50, InceptionResNetV2, MobileNet; 99.8% accuracy	CKD-FED	Focused on late-stage CKD; limited generalisability
Leung et al. (26)	RRT (renal replacement therapy) risk prediction	CNN + LSTM + ANN fusion outperforming KFRE (AUC 0.91)	CKD-LTS	No genomic or long-term longitudinal data
Pimpalkar et al. (3)	CT-image-based CKD classification	Transfer learning (ResNet50/VGG16/InceptionV3) reaching 98.5% accuracy	CKD-CT	Imaging-only dataset; lacks clinical variables
Rezk et al. (27)	Explainable AI for CKD prediction in CV patients	Applied XAI methods (e.g., SHAP) for transparency and model interpretation	CKD-CDR	Primarily ML-based; limited DL experiments
Ma et al. (28)	Kidney-failure risk prediction	KFDeep achieved AUROC 0.946 (internal) and 0.805 (external) using EHR	CKD-EHR	Lower external performance; small validation cohort
Ayogu et al. (29)	Evaluation of DL ensembles for CKD detection	Comparative evaluation showing ensemble robustness	UCI-CKD	Data imbalance; lacks standard benchmarking
Chowdhury et al. (9)	Multi-class CKD staging (clinical + genomic)	Integrated genomic + clinical features for full CKD staging	CKD-OMICS	Limited sample size; low genomic diversity
Khan et al., (30)	Early kidney disease diagnosis using hybrid DL	Used ConvNeXt + EfficientNetV2 achieving ∼96% accuracy	CKD-CDR	Pre-print; no clinical deployment evidence

## Comparative analysis and discussion

Recent studies have demonstrated the significant potential of deep learning (DL) models in improving the early detection and diagnosis of CKD. As highlighted in Table 1, various DL models such as Random Forest (RF), CNNs, and LSTMs have achieved high accuracy levels, ranging from 85% to 99% depending on the data type and model architecture. For instance, Rashed et al. (2021) reported 97% accuracy using RF, while Pimpalkar et al. (3) achieved 99.96% accuracy for CT-image-based CKD detection using fine-tuned CNN models. In Table 2, several hybrid DL models, like the ensemble model combining CNN, LSTM, and BLSTM, have been shown to improve CKD progression prediction, reaching accuracies of up to 99% in early predictions (5). The incorporation of transfer learning, such as VGG16 and ResNet50, has also played a crucial role in improving model performance, especially in small datasets. Despite these advancements, significant challenges remain. Many of these studies, as seen in Table 2, rely on small, institution-specific datasets, which limit the models' generalizability across diverse populations. Issues like class imbalance, particularly with underrepresented early-stage CKD, and poor model interpretability continue to hinder the clinical adoption of these systems (21, 29). Moreover, external validation is often lacking, which affects the robustness of these models in real-world clinical settings. Future directions should focus on multi-centre datasets, integrating clinical and imaging data, and improving explainable AI (XAI) techniques to address these challenges and enhance clinical applicability.

## Limitations and future directions

Despite promising results, current deep learning research for CKD detection faces several limitations, including the heavy reliance on small or single-centre datasets, class imbalance particularly underrepresentation of early CKD stages and limited external validation, all of which restrict the generalisability of reported findings. Model interpretability also remains a critical barrier, as many high-performing architectures function as opaque systems that offer little clinical insight into their decision processes. Future research should prioritise the development of large, multi-institutional datasets; improved handling of class imbalance through advanced sampling and synthetic data generation; and integration of multimodal information spanning imaging, laboratory time-series, clinical notes, and genomic data. Enhancing interpretability through explainable AI (XAI) frameworks will be essential for clinician trust, while privacy-preserving approaches such as federated learning can promote secure collaboration across institutions. Strengthening external and prospective validation, improving reproducibility, and aligning model development with real-world clinical workflows will be key to enabling safe, scalable adoption of deep learning systems for CKD detection.

## Conclusion

This mini-review highlights the growing role of deep learning in improving early detection and risk prediction for chronic kidney disease. Across the surveyed studies, deep learning architectures particularly CNNs, LSTMs, hybrid ensembles, and transfer-learning models consistently achieve higher accuracy and stronger diagnostic capability compared with traditional machine-learning approaches. Multi-modal integration of imaging, clinical, and laboratory features has further improved performance, demonstrating the potential of AI to identify subtle indicators of CKD and forecast disease progression earlier than standard clinical markers. However, meaningful variation remains in dataset size, data quality, validation strategies, and model interpretability, which makes direct comparison across studies challenging. Despite these limitations, the evidence clearly indicates that deep learning offers transformative opportunities for earlier diagnosis, risk stratification, and enhanced clinical decision-making in CKD.

## References

[1] Francis A Harhay MN Ong AC Tummalapalli SL Ortiz A Fogo AB Chronic kidney disease and the global public health agenda: an international consensus. Nat Rev Nephrol. (2024) 20(7):473–85. 10.1038/s41581-024-00820-6 38570631

[2] Gharaibeh M Alzu’bi D Abdullah M Hmeidi I Al Nasar MR Abualigah L Radiology imaging scans for early diagnosis of kidney tumors: a review of data analytics-based machine learning and deep learning approaches. Big Data Cogn Comput. (2022) 6(1):29. 10.3390/bdcc6010029

[3] Pimpalkar A Saini DKJB Shelke N Balodi A Rapate G Tolani M. Fine-tuned deep learning models for early detection and classification of kidney conditions in CT imaging. Sci Rep. (2025) 15(1):10741. 10.1038/s41598-025-94905-2 40155680 PMC11953426

[4] Zhang M Ye Z Yuan E Lv X Zhang Y Tan Y Imaging-based deep learning in kidney diseases: recent progress and future prospects. Insights Imaging. (2024) 15(1):50. 10.1186/s13244-024-01636-5 38360904 PMC10869329

[5] Saif D Sarhan AM Elshennawy NM. Early prediction of chronic kidney disease based on ensemble of deep learning models and optimizers. J Electr Syst Inf Technol. (2024) 11(1):17. 10.1186/s43067-024-00142-4

[6] Anoch B Parthiban L. Uncertainty-Aware AI for enhanced chronic kidney disease diagnosis: a review of explainable and reliable models. 2025 International Conference on Computational Robotics, Testing and Engineering Evaluation (ICCRTEE); 2025, May: IEEE (2025) pp. 1–6. 10.1109/iccrtee64519.2025.11053068

[7] Khan SU. Multi-level feature fusion network for kidney disease detection. Comput Biol Med. (2025) 191:110214. 10.1016/j.compbiomed.2025.110214 40233676

[8] Prasuna K Tirupatamma NL Lakshitha M Soujanya V Rakshitha B. Chronic kidney disease prediction using CNN, LSTM and ensemble model. Int J Eng Res Sci Technol. (2025) 21(2):321–30. 10.62643/

[9] Chowdhury MNH Bin Ibne Reaz M Ali SHM Crespo ML Ahmad S Salim GM Deep learning for early detection of chronic kidney disease stages in diabetes patients: a TabNet approach. Artif Intell Med. (2025) 166:103153. 10.1016/j.artmed.2025.103153 40347843

[10] Burra LR Tumuluru P Bonam J Raju SH Nalajala S Borra SPR. Innovative application of conditional deep convolutional generative adversarial networks to enhance chronic kidney disease diagnosis with uneven datasets. Smart Factories for Industry 5.0 Transformation. Beverly: Scrivener Publishing (2025). p. 71–87. 10.1002/9781394200467.ch4

[11] Kanagamalliga S Gayathri VR. Recent advances in deep learning-based chronic kidney disease detection. 2025 6th International Conference on Intelligent Communication Technologies and Virtual Mobile Networks (ICICV); 2025, June IEEE. (2025). p. 692–7. 10.1109/icicv64824.2025.11085587

[12] Poudel S Bhatt LP Prasad PC Chandra Paudel A. Prediction of chronic kidney disease using random forest, XGBoost and ANN model. J Adv Coll Eng Manage. (2025) 10:121–33. 10.3126/jacem.v10i1.76323

[13] Hsu C-T Huang C-Y Chen C-H Deng Y-L Lin S-Y Wu M-J. Machine learning models to predict osteoporosis in patients with chronic kidney disease stage 3–5 and end-stage kidney disease. Sci Rep. (2025) 15(1):11391. 10.1038/s41598-025-95928-5 40181057 PMC11968914

[14] Ramesh JVN Lakshmi PNS Syamsundararao T Muniyandy E Ushasree L El-Ebiary YAB Enhancing chronic kidney disease prediction with deep separable convolutional neural networks. Int J Adv Comput Sci Appl. (2025) 16(2). 10.14569/ijacsa.2025.01602100

[15] Zhu Y Bi D Saunders M Ji Y. Prediction of chronic kidney disease progression using recurrent neural network and electronic health records. Sci Rep. (2023) 13(1):22091. 10.1038/s41598-023-49271-2 38086905 PMC10716428

[16] Pinto A Ferreira D Neto C Abelha A Machado J. Data mining to predict early stage chronic kidney disease. Procedia Comput Sci. (2020) 177:562–7. 10.1016/j.procs.2020.10.079

[17] Swamy BN Nakka R Sharma A Praveen SP Thatha VN Gautam K. An ensemble learning approach for detection of chronic kidney disease (CKD). J Intell Syst Internet Things. (2023) 10(2):38–48. 10.54216/jisiot.100204

[18] Almansour NA Syed HF Khayat NR Altheeb RK Juri RE Alhiyafi J Neural network and support vector machine for the prediction of chronic kidney disease: a comparative study. Comput Biol Med. (2019) 109:101–11. 10.1016/j.compbiomed.2019.04.017 31054385

[19] Ilyas H Ali S Ponum M Hasan O Mahmood MT Iftikhar M Chronic kidney disease diagnosis using decision tree algorithms. BMC Nephrol. (2021) 22(1):273. 10.1186/s12882-021-02474-z 34372817 PMC8351137

[20] Ramu K Patthi S Prajapati YN Ramesh JVN Banerjee S Rao KBVB Hybrid CNN-SVM model for enhanced early detection of chronic kidney disease. Biomed Signal Process Control. (2025) 100:107084. 10.1016/j.bspc.2024.107084

[21] Hegde GM Shenoy PD Venugopal KR Canchi A. A deep learning framework for chronic kidney disease stage classification. Healthc Anal. (2025) 7:100398. 10.1016/j.health.2025.100398

[22] Rahman MH Rahaman M Arafat Y Islam U Hasan R Hossain T Artificial intelligence for chronic kidney disease risk stratification in the USA: ensemble vs. Deep learning methods. Br J Nurs Stud. (2025) 5(2):20–32. 10.32996/bjns.2025.5.2.3

[23] Batra A Chatterjee P Chaki J. Federated learning with AutoAlbum for kidney disease detection: a privacy-preserving approach to medical image analysis. IEEE Access. (2025). 10.21227/646s-6y35

[24] Simeri A Pezzi G Arena R Papalia G Szili-Torok T Greco R Artificial intelligence in chronic kidney diseases: methodology and potential applications. Int Urol Nephrol. (2024) 57(1):159–68. 10.1007/s11255-024-04165-8 39052168 PMC11695560

[25] Canbay Y Adsiz S Canbay P. Privacy-Preserving transfer learning framework for kidney disease detection. Appl Sci. (2024) 14(19):8629. 10.3390/app14198629

[26] Leung K-C Ng WW-S Siu Y-P Hau A K C Lee H-K. Deep learning algorithms for predicting renal replacement therapy initiation in CKD patients: a retrospective cohort study. BMC Nephrol. (2024) 25(1):95. 10.1186/s12882-024-03538-6 38486160 PMC10938811

[27] Rezk NG Alshathri S Sayed A Hemdan EE. Explainable AI for chronic kidney disease prediction in medical IoT: integrating GANs and few-shot learning. Bioengineering. (2025) 12(4):356–356. 10.3390/bioengineering12040356 40281716 PMC12025083

[28] Ma J Wang J Lu L Sun Y Feng M Zhang F *Development and Validation of a Dynamic Kidney Failure Prediction Model based on Deep Learning: A Real-World Study with External Validation. ArXiv.org* (2025). Available online at: https://arxiv.org/abs/2501.16388 (Accessed September 25, 2025).

[29] Ayogu II Daniel CF Ayogu BA Odii JN Okpalla CL Nwokorie EC. Investigation of ensembles of deep learning models for improved chronic kidney diseases detection in CT scan images. Franklin Open. (2025) 11:100298. 10.1016/j.fraope.2025.100298

[30] Khan ZA Waqar M Khan HU Chaudhary NI Khan AT Ishtiaq I Fine-tuned deep transfer learning: an effective strategy for the accurate chronic kidney disease classification. PeerJ Computer Science. (2025) 11:e2800. 10.7717/peerj-.cs.2800 40567689 PMC12190387

[31] Rashed SH Maher FT Al-Helaly LA. Purification and characterization of meprin a from human blood serum. Ann Rom Soc Cell Biol. (2021) 25(2):4209–20. http://annalsofrscb.ro/index.php/journal/article/view/1440

[32] Ravizza S Huschto T Adamov A Böhm L Büsser A Flöther FF Predicting the early risk of chronic kidney disease in patients with diabetes using real-world data. Nat Med. (2019) 25(1):57–9. 10.1038/s41591-018-0239-8 30617317

[33] Xiong C Wu Q Fang M Li H Chen B Chi T. Protective effects of luteolin on nephrotoxicity induced by long-term hyperglycaemia in rats. J Int Med Res. (2020) 48(4):0300060520903642. 10.1177/0300060520903642 32242458 PMC7132816

